# Application of pea-like yolk–shell structured Fe_3_O_4_@TiO_2_ nanosheets for photocatalytic and photo-Fenton oxidation of bisphenol-A†

**DOI:** 10.1039/c9ra04084f

**Published:** 2019-07-17

**Authors:** Xingxing Li, Mingcan Cui, Yonghyeon Lee, Jongbok Choi, Jeehyeong Khim

**Affiliations:** School of Civil, Environmental, and Architectural Engineering, Korea University 145 Anam-ro Seongbuk-gu Seoul 02841 Republic of Korea hyeong@korea.ac.kr

## Abstract

Uniform pea-like yolk–shell (PLYS) structured magnetic TiO_2_(PLYS-Fe_3_O_4_@TiO_2_) nanosheets have been prepared *via* a combined kinetics-controlled mechanical force-driven and hydrothermal etching assisted crystallization method and characterized. The resulting PLYS-Fe_3_O_4_@TiO_2_ nanosheets possess well defined yolk–shell structures with a large BET surface area (∼187.26 m^2^ g^−1^) and a strong magnetic susceptibility (∼17.4 emu g^−1^). The reaction rate constant was 24.2 × 10^−2^ min^−1^ as a result of oxidative decomposition of BPA using UV/PLYS-Fe_3_O_4_@TiO_2_/H_2_O_2_ system. This is 1.1 and 8.34 times faster than the BPA decomposition reaction rate constant in UV/TiO_2_/H_2_O_2_ and UV/Fe_3_O_4_/H_2_O_2_ systems, respectively. The synthesized catalyst also exhibited excellent recycle capability and excellent acid decomposition performance.

## Abstract 2

Uniform pea-like yolk–shell (PLYS) structured magnetic TiO_2_(PLYS-Fe_3_O_4_@TiO_2_) nanosheets have been prepared *via* a combined kinetics-controlled mechanical force-driven and hydrothermal etching assisted crystallization method and characterized.

## Graphical abstract

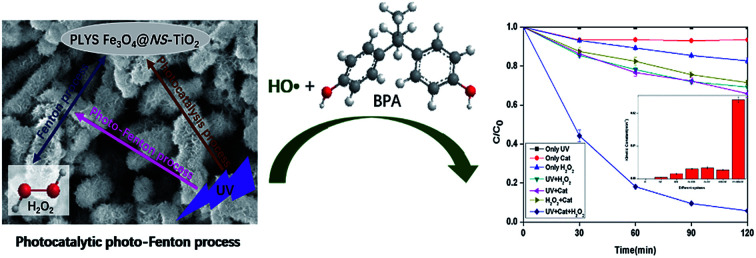

## 1. Introduction

Emerging organic contaminants (EOCs) are a burgeoning and extremely diverse class of contaminants that are not routinely monitored but have the great potential to enter the environment and may cause known or suspected adverse ecological and human health effects. EOCs of major concern include endocrine disrupting chemicals, pharmaceuticals and personal care products, surfactants, and various industrial additives as well as hormones. BPA, classified as an endocrine disruptor, is a contaminant of importance because it is extensively used in the production of polycarbonates, epoxy resins, and other plastics.^1^ Due to the low concentration, high chemical stability and low biodegradability of BPA, the effectiveness of conventional treatment technologies such as adsorption, membrane filtration, and biological treatment are typically limited.^2^ As an alternative, advanced oxidation processes (AOPs) have been demonstrated as a highly efficient technology for the destruction and mineralization of organic pollutants through powerful reactive oxidation species such as hydroxyl radicals (HO˙).^3–6^ Among various AOPs, homogeneous Fenton reaction based on ferrous ions and hydrogen peroxide is of great importance owing to the environmentally friendly characteristics. However, the narrow working pH range, difficulties to recover the dissolved metal ions and necessity for further treatment of ferric hydroxide sludge greatly hinder its wide application for practical water treatment.^7,8^ To this end, heterogeneous Fenton process is developed as a valid approach to overcome these kinds of drawbacks.

Recently, Nemanja *et al.*^9^ prepared an effective photo-Fenton catalyst of Fe/TiO_2_ by deposition–precipitation method for degradation of thiacloprid. However, the catalyst separation issue seems to be a big challenge. Although Lejin *et al.*^10^ synthesized Fe_3_O_4_ magnetic particles *via* the co-precipitation of Fe^2+^ and Fe^3+^ method and observed the effect of different parameters, the degradation efficiency of 2,4-dichlorophenol is not high. Xiaoliang *et al.*^11^ demonstrated the heterogeneous Fenton-like process for the treatment of methylene blue (MB), whereas the core–shell structured Fe_3_O_4_@C nanoparticles only worked well in acidic environment. Sheng-Tao *et al.*^12^ showed the core–shell structured Fe_3_O_4_@SiO_2_ nanoparticles as efficient Fenton-like catalyst in neutral environment for the degradation of MB but the mechanism still requires farther investigations. In addition, yolk–shell material with a distinctive core@void@shell configuration, has stimulated considerable interest because of the void space between the core and the shell which can provide as a reactor. Dan *et al.*^13^ reported that yolk–shell structured Fe_3_O_4_@TiO_2_ nanoparticles as a high-performance catalyst for the combination of photo-Fenton degradation of tetracycline. However, due to Fe_3_O_4_ was fully covered by the TiO_2_ shell, it can cause lots of disadvantages.

In this study, we design and prepare an advanced PLYS-Fe_3_O_4_@TiO_2_ nanosheets as a heterogeneous photocatalytic photo-Fenton catalyst. Instead of TiO_2_ shell, TiO_2_ nanosheets are coated which cannot only increase the surface area, but also shorter the penetration pathway of both H_2_O_2_ and light. Most importantly, even after 3 times cycle test, the catalyst still shows high activity for the degradation of BPA under a neutral condition.

## 2. Materials and methods

### 2.1 Reagents

Iron chloride hexahydrate (FeCl_3_·6H_2_O), sodium citrate tribasic dehydrate, sodium acetate (NaAc), bisphenol-A, concentrated ammonia solution (28 wt%), *t*-butanol, titanium(iv) isopropoxide (TIPO), TiO_2_ (P25, 20% rutile and 80% anatase) and tetraethyl orthosilicate (TEOS) were analytical grade and purchased from Sigma-Aldrich (USA). Sodium hydroxide (NaOH), hydrochloric acid (HCl, 36%), perchloric acid (HClO_4_), potassium bi-phthalate (C_6_H_4_COOKCOOH, 99.7%), ammonium molybdate ((NH_4_)_6_Mo_7_O_24_·4H_2_O, 99.0%), ethylene glycol and ethanol were purchased from Samchun Pure Chemicals. All chemicals were analytical grade and used as received without further purification. Corp. deionized water was used for all experiments.

### 2.2 Preparation of catalysts

#### 2.2.1 Synthesis of Fe_3_O_4_ nanoparticles

The superparamagnetic Fe_3_O_4_ nanoparticles were prepared *via* a solvothermal method reported previously.^14^ Briefly, FeCl_3_·6H_2_O (3.25 g), sodium citrate tribasic dehydrate (1.3 g), and sodium acetate (NaAc, 6.0 g) were dissolved in ethylene glycol (80 mL) with agitation. The mixture was stirred vigorously for 1 h at room temperature and then transferred into a Teflon-lined stainless-steel autoclave (100 mL). The autoclave was heated at 200 °C for 10 h, and then allowed to cool to room temperature. The black products were washed with deionized water and ethanol for 3 times, respectively.

#### 2.2.2 Synthesis of Fe_3_O_4_@SiO_2_@TiO_2_ nanoparticles

The core–shell Fe_3_O_4_@SiO_2_ nanospheres were prepared according to a Stöber sol–gel method.^12,15^ For a typical synthesis, an ethanol dispersion of the Fe_3_O_4_ magnetite particles obtained above (6.0 mL, 0.05 g mL^−1^) was added to a three-neck round-bottom flask with ethanol (70 mL), deionized water (30 mL) and concentrated ammonia solution (2.0 mL, 28 wt%). The mixed solution was sonicated for 20 min. Then, 1.0 mL of TEOS was added dropwise in 5 min, and the reaction was allowed to proceed for 1 h under continuous mechanical stirring at room temperature. The resultant products (denoted as Fe_3_O_4_@SiO_2_) were separated and collected with a magnet, followed by washing with deionized water and ethanol for 3 times, respectively.

The as-prepared Fe_3_O_4_@SiO_2_ nanospheres were further coated with a TiO_2_ shell through a kinetic-controlled Stöber method. Briefly, the core–shell Fe_3_O_4_@SiO_2_ nanospheres (0.1 g) were dispersed in ethanol (100 mL), and mixed with concentrated ammonia solution (0.80 mL, 28 wt%) under ultrasound for 15 min. Subsequently, 0.75 mL of TIPO was added dropwise in 5 min, and the reaction was allowed to proceed for 24 h at 45 °C under continuous mechanical stirring. The resultant products (denoted as pea-like core–shell Fe_3_O_4_@SiO_2_@TiO_2_) were separated with a magnet and washed with deionized water and ethanol for 3 times, respectively.

#### 2.2.3 Synthesis of PLYS-Fe_3_O_4_@TiO_2_

The PLYS-Fe_3_O_4_@TiO_2_ nanoparticles were prepared through an alkaline hydrothermal etching assisted crystallization method. The above obtained Fe_3_O_4_@SiO_2_@TiO_2_ nanoparticles (1.0 g) were mixed with NaOH solution (30 mL, 1.0 M), then transferred into a Teflon-lined stainless-steel autoclave (100 mL in capacity). The autoclave was heated at 200 °C for 24 h and then cooled to room temperature. The products were collected by a magnet and added in HCl solution (50 mL, 0.1 M) for 15 min, washed with deionized water until pH value was around 7 and subsequently dried at 60 °C thoroughly in vacuum oven.

The resultant products (denoted as PLYS-Fe_3_O_4_@TiO_2_) were calcined 400 °C in N_2_ atmosphere for 2 h, then the PLYS-Fe_3_O_4_@TiO_2_ nanosheets were obtained.

### 2.3 Materials characterization

X-ray diffraction (XRD) patterns were recorded on a Bruker D8X-ray diffractometer with Ni-filtered Cu Kα radiation (40 kV, 40 mA). Nitrogen sorption isotherms were measured at 77 K with a Micromeritics Tristar 3020 analyzer (USA). Prior to measurements, the samples were degassed in a vacuum at 180 °C for 6 h. The Brunauer–Emmett–Teller (BET) method was utilized to calculate the specific surface areas (*S*_BET_) using adsorption data in the relative pressure range *P*/*P*_0_ = 0.04–0.2. Using the Barrett–Joyner–Halenda (BJH) model, the pore size distributions were derived from the adsorption branches of the isotherms, and the total pore volumes (*V*) were estimated from the adsorbed amount at the relative pressure *P*/*P*_0_ = 0.995. Transmission electron microscopy (TEM) was carried out on a JEOL 2011 microscope (Japan) operated at 200 kV. For TEM measurements, the sample was suspended in ethanol and supported on a holey carbon film on a Cu grid. High-resolution transmission electron microscopy (HRTEM) observations were performed on JEM-2100F transmission electron microscope with an accelerating voltage of 200 kV equipped with a post-column Gatan imaging filter (GIF-Tri-dium). Scanning electron microscopy (SEM) images were taken using a Zeiss ultra 55 ultrahigh resolutions thermal FEG with an in-lens electron optic operating at 3 kV. The magnetization was measured using a Vibrating Sample Magnetometer (EV9 including automatic sample rotation, Microsense, Japan) under a magnetic field of 10 kOe and a temperature of 24 °C.

### 2.4 Experimental procedure

All experiments were conducted in cylindrical batch reactors (*Φ*2.0 × 10.0 cm, total volume: 50 mL) with a shaking water bath to mix and maintain the temperature (Fig. S1†). A thermometer (Tecpel DTM-318) was used to measure the temperature. The light source (Fig. S2†) was HANSUNG G12T5 UV lamp (*λ* = 254 nm, 5 × 10 W) and the light intensity in the centre of the BPA solution was 800 μW cm^−2^. The reaction suspension was prepared by adding appropriate amounts of catalyst and H_2_O_2_ into 30 mL BPA solution. The desired pH value was adjusted by HClO_4_ or NaOH and measured by a pH meter (Orion 3 Star). Prior to addition of H_2_O_2_, the mixture was mixed in dark for 30 min to reach the adsorption/desorption equilibrium between the catalyst and pollutants. Afterwards, 1.0 mL of the suspension was removed using a 2 mL syringe at given time intervals and filtered *via* a membrane with a pore size of ∼0.45 mm. Furthermore, 10 μL 0.5 M *n*-butanol was added to the sample above to terminate the reaction and the BPA concentration in each sample was analysed on a high-performance liquid chromatography (Agilent 1260) with an Eclipse XDB C18 column (4.6 × 250 mm, 5 μm) and a diode array UV detector (G4212B 1260 DAD, *λ* = 210 nm). For the reusability test, the catalysts were collected by magnetic separation, washed with deionized water several times, dried in vacuum and used it for the next reaction under similar experimental conditions. Experiments were carried out 3 times and all results were expressed as a mean value. The total organic carbon (TOC) was determined with a laboratory TOC analyzer (SIEVERS 5310C). Leached iron ions were detected by an Inductively Coupled Plasma Atomic Emission Spectrometer (ICP-AES; PerkinElmer 5300DV).

## 3. Results and discussion

### 3.1 Synthesis methods and characterizations

**Fig. 1:**
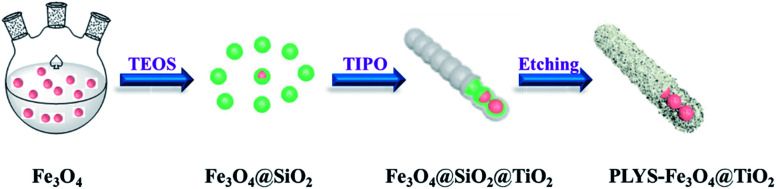
Schematic illustration of the formation process of the PLYS-Fe_3_O_4_@TiO_2_ nanoparticles.

Fig. 1 illustrates the diagrammatic sketch of the synthesis procedure which involves three steps. In step (1), the pre-synthesized magnetic Fe_3_O_4_ nanoparticles were coated with silica layer *via* sol–gel method using TEOS as a precursor. In step (2), a further sol–gel process was used to coat TiO_2_ shell on the silica layer using TIPO as the precursor. In step (3), after a hydrothermal etching method followed by calcination process, PLYS-Fe_3_O_4_@TiO_2_ nanosheets were formed.

**Fig. 2:**
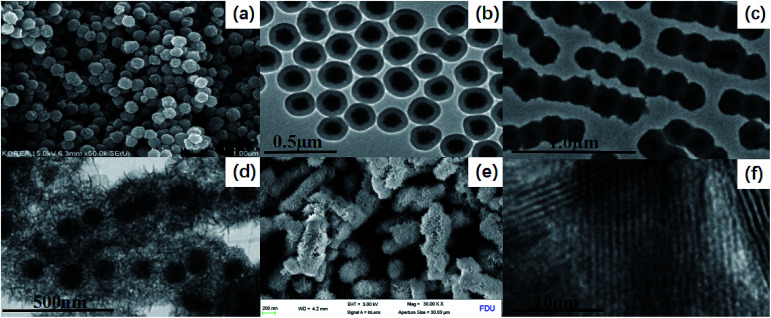
SEM and TEM images: (a) SEM images of Fe_3_O_4_ nanoparticles, (b) TEM images of Fe_3_O_4_@SiO_2_ nanospheres, (c) TEM image Fe_3_O_4_@SiO_2_@TiO_2_, (d) TEM images of PLYS-Fe_3_O_4_@TiO_2_, (e) SEM image PLYS-Fe_3_O_4_@TiO_2_, (f) HRTEM image of the TiO_2_ nanosheet.

The uniform magnetite particles can be prepared through a facile solvothermal reaction based on a high temperature reduction of Fe(iii) salts with ethylene glycol in the presence of trisodium citrate. SEM images clearly reveal that the obtained Fe_3_O_4_ particles possess a uniform spherical shape with an average diameter of ∼130 nm (Fig. 2(a)). The particles exhibit excellent dispersibility in polar solvents such as water and ethanol because of numerous citrate groups anchored on the surface, facilitating the subsequent coating with silica and titania. The PLYS-Fe_3_O_4_@TiO_2_ nanospheres after the first sol–gel process show a relatively smooth surface with a diameter of ∼180 nm (Fig. 2(b)). TEM images reveal that a silica layer with a thickness of ∼40 nm is uniformly coated onto the magnetic core, resulting in a well-defined core–shell structure.

The further sol–gel process leads to the formation of the pea-like Fe_3_O_4_@SiO_2_@TiO_2_ particles with an average thickness of TiO_2_ layer is ∼120 nm (Fig. 2(c)). After the hydrothermal treatment, the PLYS-Fe_3_O_4_@TiO_2_ nanoparticles with a uniform size of 900 nm are obtained (Fig. 2(d)). As represented in Fig. 2(e), PLYS-Fe_3_O_4_@TiO_2_ nanoparticles possess a unique yolk–shell structure that the diameters of the inner and of the outer layer are 200 and 320 nm. Fig. 2(f) clearly indicates that the lattice fringes of the nanosheet are 0.35 nm which can be analogous to the (101) planes of anatase TiO_2_.^16^

**Fig. 3:**
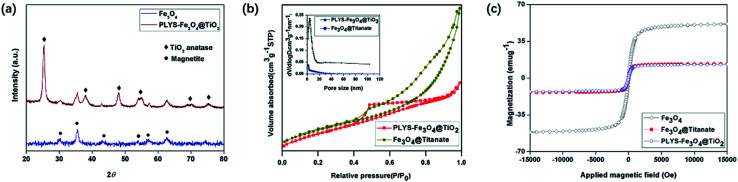
(a) XRD patterns of Fe_3_O_4_ and PLYS-Fe_3_O_4_@TiO_2_, (b) N_2_ sorption isotherm of PLYS-Fe_3_O_4_@TiO_2_ and Fe_3_O_4_@Titanate, (c) magnetic hysteresis loops of Fe_3_O_4_, Fe_3_O_4_@Titanate and PLYS-Fe_3_O_4_@TiO_2_ at 25 °C.

The X-ray diffraction (XRD) pattern of PLYS-Fe_3_O_4_@TiO_2_ (Fig. 3(a)) shows six well resolved characteristic diffraction peaks of (320), (331), (400), (422), (511), (440), which are typical for Fe_3_O_4_ crystalline phase.^17^ In addition, new characteristic peaks can be clearly distinguished compared with XRD pattern of pure Fe_3_O_4_ nanoparticles.

N_2_ sorption isothermal (Fig. 3(b)) shows that the PLYS-Fe_3_O_4_@TiO_2_ nanoparticles have a nanoporous structure and the BET surface area is calculated to be 187.26 m^2^ g^−1^ which is a little decrease compared with PLYS-Fe_3_O_4_@TiO_2_ (208.38 m^2^ g^−1^). The integrated energy dispersive X-ray spectroscopy (EDS) analysis of PLYS-Fe_3_O_4_@TiO_2_ (Fig. S8†) further confirms the presence of elements Ti, Fe and O. In addition, the Fe content is ∼13.17 wt%.

Meanwhile, the saturation magnetization value of pure Fe_3_O_4_ and PLYS-Fe_3_O_4_@TiO_2_ nanoparticle (Fig. 3(c)) were measured to be 51.7 and 17.4 emu g^−1^ respectively, which could be ascribed to the existence of TiO_2_ nanosheets.

Furthermore, to observe the mechanism of the formation of PLYS-Fe_3_O_4_@TiO_2_ double-shelled yolk–shell microspheres, a series of experiments were carried out. With the change of ammonia content (0.4, 0.5, 0.6 and 0.7 mL), the typical sandwich sphere structure was converted to a pea-like yolk–shell structure (Fig. S3†). And using different diameter of mixing paddle (4, 5 and 6 cm), the length of pea-like particles was changed (Fig. S4†) with different concentration of sodium hydroxide solution, the structure of PLYS-Fe_3_O_4_@TiO_2_ spheres had a big change (Fig. S6†), but there was no big difference for PLYS-Fe_3_O_4_@TiO_2_ particles. However, the PLYS-Fe_3_O_4_@TiO_2_ double-shelled structure will be collapsed under the high concentration of sodium hydroxide (Fig. S7A†). In addition, with higher concentration of hydrochloric acid, the pea-like yolk–shell structure would be changed (Fig. S7B†).

On the basis of the above observations, we propose a combination of kinetics-controlled mechanical force-driven growth (Fig. S5†) and hydrothermal etching assisted crystallization method for the formation of the PLYS-Fe_3_O_4_@TiO_2_ double-shelled yolk–shell nanoparticles. Because of the high initial ammonia content, the heterogeneous and homogenous nucleation processes simultaneously occur^18^ and the pea-like core–shell Fe_3_O_4_@SiO_2_@TiO_2_ nanoparticles can be formed under the mechanical force.^19,20^ Subsequently, the amorphous silica layer is etched by NaOH solution first, then the alkali solution can permeate and etch porous TiO_2_ shell to form two layers titanate nanosheets.^21^

### 3.2 Catalytic activity of PLYS-Fe_3_O_4_@TiO_2_

#### 3.2.1 Degradation of BPA under different systems

**Fig. 4:**
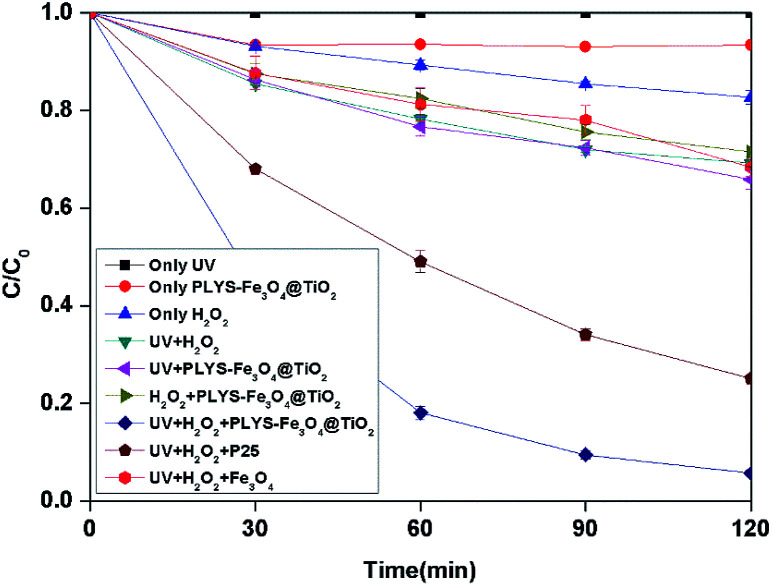
The curves of different systems with reaction time in photocatalytic photo-Fenton degradation of BPA at fixed [H_2_O_2_] = 18.9 mM, [PLYS-Fe_3_O_4_@TiO_2_] = 1.5 g L^−1^, [BPA] = 0.088 mM, pH = 7, light intensity = 800 μW cm^−2^, *T* = 25 °C.

As shown in Fig. 4, BPA was used as a model pollutant to investigate the catalytic activity under different conditions within 2 h. According to the results, 0%, 17.3% and 6.5% BPA can be removed by only UV, H_2_O_2_ and catalyst in the dark independently. After being irradiated, the concentration of BPA decreases to 65.9%, 54.5% and 5.7% in UV/PLYS-Fe_3_O_4_@TiO_2_/H_2_O_2_, UV/TiO_2_(P25)/H_2_O_2_, UV/H_2_O_2_/Fe_3_O_4_, system, respectively.

Furthermore, the reaction kinetic constants were evaluated through fitting the experimental data with Langmuir–Hinshelwood model to better compare the catalytic performance. And the degradation kinetic curves can be assumed as pseudo first-order kinetic eqn (1).

1 −ln(*C*/*C*_0_) = *kt* + *b*

where *C*_0_ is the initial concentration, *C* is the concentration at time *t*, *k* is the apparent rate constant, respectively.

Fig. 4 shows the BPA decomposition reaction rate constants in different systems related to this study. Table S1† Comparing reaction rate constants in different systems and conditions.

The kinetic constant value of UV/H_2_O_2_/PLYS-Fe_3_O_4_@TiO_2_ is 24.2 × 10^−3^ min^−1^ higher than the summation of UV/PLYS-Fe_3_O_4_@TiO_2_ (3.4 × 10^−3^ min^−1^), UV/TiO_2_/H_2_O_2_ (11.5 × 10^−3^ min^−1^), UV/Fe_3_O_4_/H_2_O_2_ (2.9 × 10^−3^ min^−1^), UV/H_2_O_2_ (3.0 × 10^−3^ min^−1^) and H_2_O_2_/PLYS-Fe_3_O_4_@TiO_2_ (2.7 × 10^−3^ min^−1^).

The results indicate that the relatively poor adsorption efficiency of BPA on the PLYS-Fe_3_O_4_@TiO_2_ catalysts and the BPA oxidation capacity of H_2_O_2_ directly is weaker than hydroxyl radical. It also suggests that the synergetic effect between photocatalytic process and photo-Fenton process, which not only inhibits the recombination between electrons and holes, but also accelerates the reaction speed of Fe^3+^ to Fe^2+^ to increase the kinetic constant. This is similar to the value given in the ref. 22 and 23.

#### 3.2.2 Effect of H_2_O_2_ dose

**Fig. 5:**
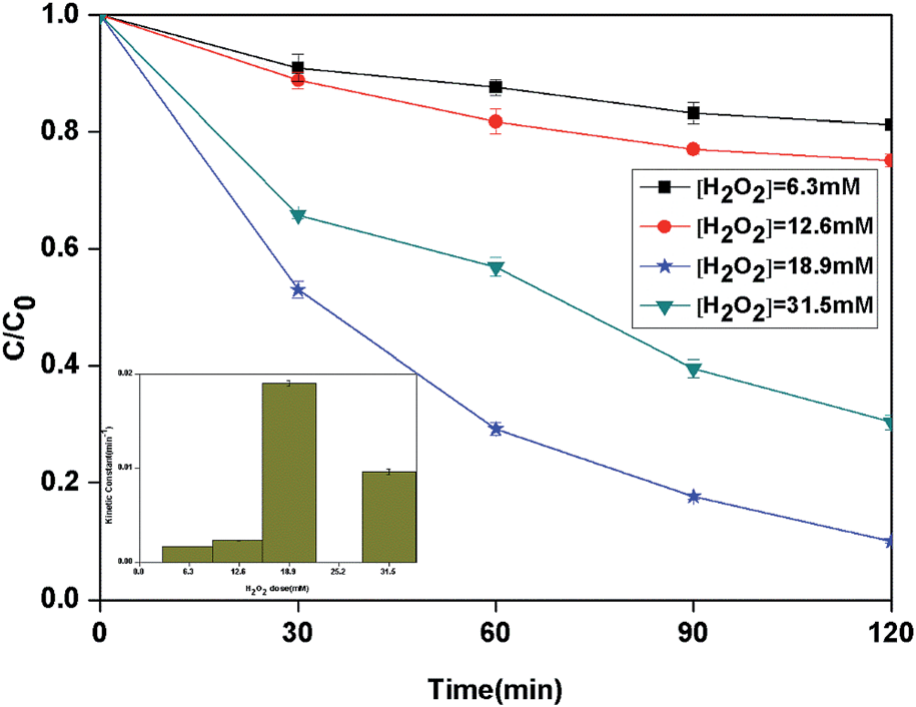
The curves of different H_2_O_2_ dose with reaction time in photocatalytic photo-Fenton degradation of BPA at fixed [PLYS-Fe_3_O_4_@TiO_2_] = 1.0 g L^−1^, [BPA] = 0.088 mM, pH = 7, light intensity = 800 μW cm^−2^, *T* = 25 °C.

Hydrogen peroxide plays the important role of BPA degradation in heterogeneous photo-Fenton reaction. The effect of different initial H_2_O_2_ dose on the degradation of BPA in the heterogeneous photocatalytic photo-Fenton process was investigated (Fig. 5). When the H_2_O_2_ dose was increased from 6.3 to 18.9 mM, the kinetic constant of BPA degradation increased from 1.7 × 10^−3^ to 19.0 × 10^−3^ min^−1^ correspondingly. However, the further increase of H_2_O_2_ dose to 31.5 mM led to a decrease of kinetic constant to 9.6 × 10^−3^ min^−1^. Theoretically, 72 mol of H_2_O_2_ are needed to completely degrade 1 mol of the BPA (eqn (2)–(4)). From the results, it can be seen that the maximum degradation occurred with H_2_O_2_ to BPA molar ratio which is 3 times as large as stoichiometric ratio.

2 C_15_H_16_O_2_ + 72HO˙ → 15CO_2_ + 44H_2_O

3 Fe^2+^ + H_2_O_2_ → Fe^3+^ + HO˙ + OH^−^

4 C_15_H_16_O_2_ + 72H_2_O_2_ → 15CO_2_ + 44H_2_O

The enhancement of degradation rate is because of the increase in HO˙.^24,25^ Moreover, further addition of H_2_O_2_ dose did not improve the degradation efficiency, which may be explained by the scavenging effect of HO˙ at a higher H_2_O_2_ dose eqn (5).^26,27^5
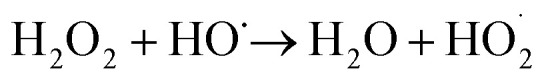


#### 3.2.3 Effect of catalyst dose

**Fig. 6:**
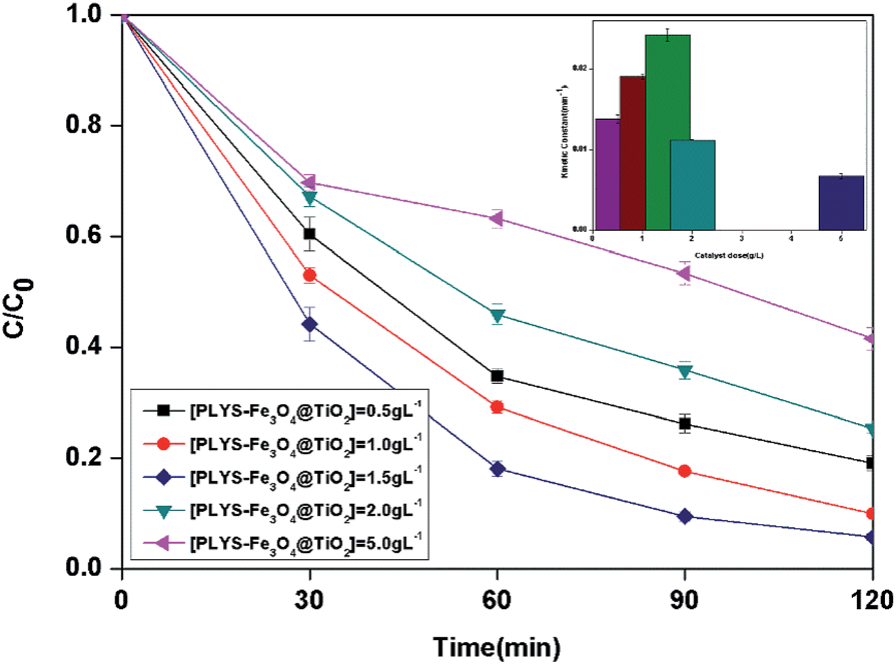
The curves of different catalyst dose with reaction time in photocatalytic photo-Fenton degradation of BPA at fixed [H_2_O_2_] = 18.9 mM, [BPA] = 0.088 mM, pH = 7, light intensity = 800 μW cm^−2^, *T* = 25 °C.

The BPA concentration change with catalyst dose from 0.5 to 5.0 g L^−1^ and the pseudo first order reaction rate constants are shown in Fig. 6. The maximum kinetic constant (24.2 × 10^−3^ min^−1^) was obtained with 1.5 g L^−1^, which is about 75.4% greater than the kinetic constant with 0.5 g L^−1^ (13.8 × 10^−3^ min^−1^). Furthermore, there is a decrease of kinetic constant which was 6.8 × 10^−3^ min^−1^ when the catalyst dose was 5.0 g L^−1^.

The increase of the degradation rate might be attributed to a number of active sites on the surface of both Fe_3_O_4_ and TiO_2_, which cannot only be occupied by H_2_O_2_, but also enhance the light utilization to generate more hydroxyl radicals. The decrease of kinetic constant after 1.5 g L^−1^ might be due to three reasons, higher turbidity which can inhibit the further penetration of light into the reactor, the consuming of HO˙ by excess Fe^2+^ and other radicals, such as 
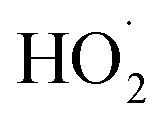
eqn (6) and (7)^28^ and catalyst agglomeration.^29^

6 Fe^2+^ + HO˙ → Fe^3+^ + OH^−^

7
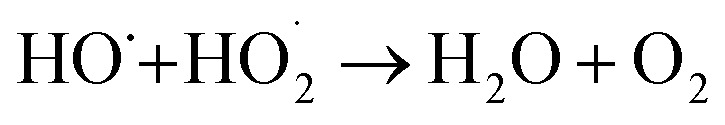


#### 3.2.4 Effect of pH

**Fig. 7:**
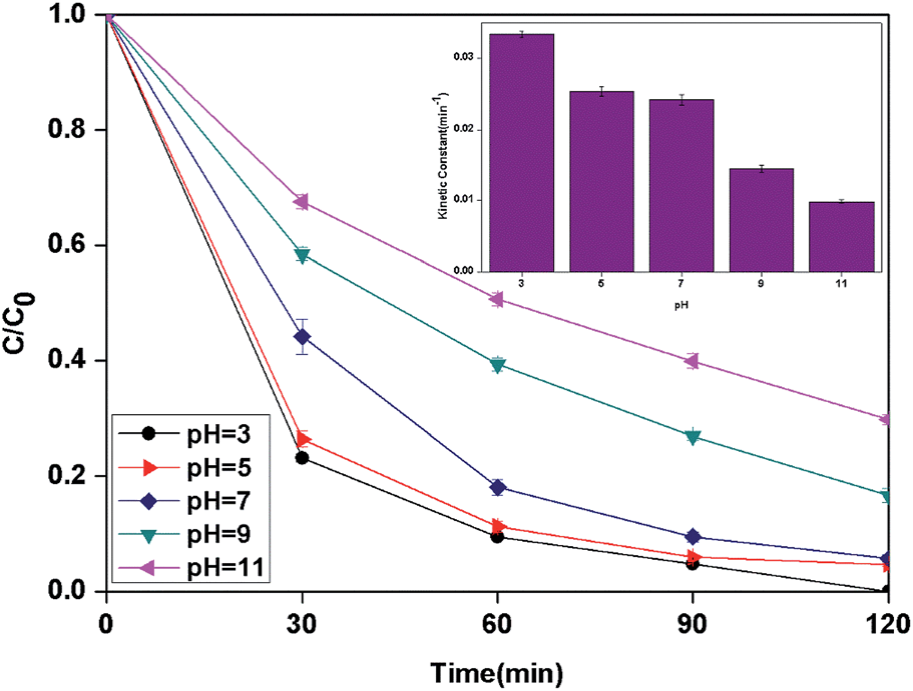
The curves of different pH with reaction time in photocatalytic photo-Fenton degradation of BPA at fixed [PLYS-Fe_3_O_4_@TiO_2_] = 1.5 g L^−1^, [H_2_O_2_] = 18.9 mM, [BPA] = 0.088 mM, light intensity = 800 μW cm^−2^, *T* = 25 °C.

It is well known that the pH value is an important parameter in the photocatalytic process. The effect of initial pH on BPA degradation in a range of 3 to 11 is shown in Fig. 7, which shows that the maximum kinetic constant at pH 3 is 33.4 × 10^−3^ min^−1^. As the pH was increased from pH 5 to pH 11, the reaction rate constants were decreased to 25.4 × 10^−3^, 14.5 × 10^−3^ and 9.9 × 10^−3^ min^−1^, respectively. This may be attributed to that pH affects TiO_2_ through the charge (eqn (8) and (9)) on the particle surface and aggregation size which can cause the change of specific surface area and light absorption.^30,31^ On the other side, the increased oxidation efficiency at lower pH values can be due to the higher oxidation potential of hydroxyl radicals. Based on eqn (10) and (11) (Nernst equation), the redox potential of HO˙/H_2_O at pH 3, 5, 7, 9 and pH 11, are 2.623 V, 2.505 V, 2.387 V, 2.269 and 2.151 V, respectively. Meanwhile, H_2_O_2_ is more stable in acidic condition than alkaline condition (eqn (12)).

8 TiOH + H^+^ → TiOH^2+^

9 TiOH + OH^−^ →TiO^−^ + H_2_O

10
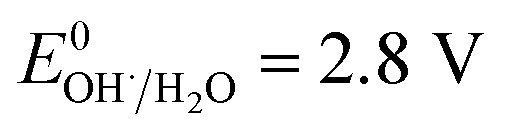
11
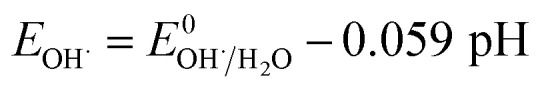

12 H_2_O_2_ ↔ H^+^ + HO^2−^

In addition, it should be noticed that catalytic activity of PLYS-Fe_3_O_4_@TiO_2_ was slightly affected by pH values from 5 to 7. This phenomenon is important because one of the major drawbacks of Fenton reaction is the narrow range of pH.

#### 3.2.5 Effect of initial concentration of BPA

**Fig. 8:**
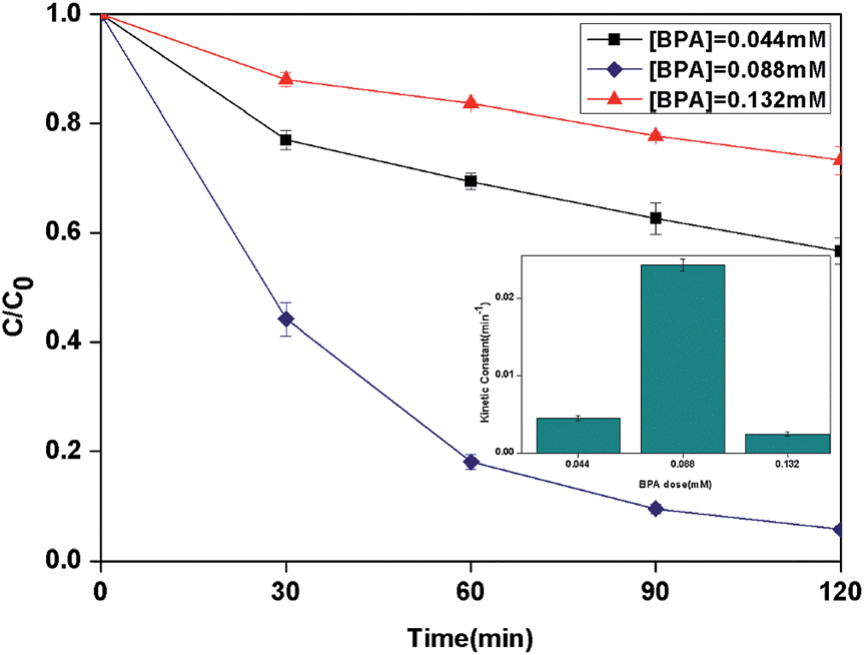
The curves of different initial BPA concentration with reaction time in photocatalytic photo-Fenton degradation of BPA at fixed [PLYS-Fe_3_O_4_@TiO_2_] = 1.5 g L^−1^, [H_2_O_2_] = 18.9 mM, pH = 7, light intensity = 800 μW cm^−2^, *T* = 25 °C.

The effect of the initial concentration of BPA on the photocatalytic photo-Fenton degradation was evaluated from 0.044 to 0.132 mM as shown in Fig. 8. The kinetic constant was increased from 4.5 × 10^−3^ to 24.2 × 10^−3^ min^−1^ with the increase of the initial concentration from 0.044 to 0.088 mM. When the initial concentration increased to 0.132 mM, the kinetic constant decreased to 2.5 × 10^−3^ min^−1^.

This phenomenon may be related to the fact that the excessive dose of H_2_O_2_ and catalyst for lower initial concentration of BPA which leading to the scavenging effect of HO˙.^32^ Furthermore, with the increasing of BPA concentration, more BPA molecules could be adsorbed on the surface of catalyst which can form the blocked active sites to decease the generation of HO˙.^33,34^

### 3.3 Mechanism investigation

**Fig. 9:**
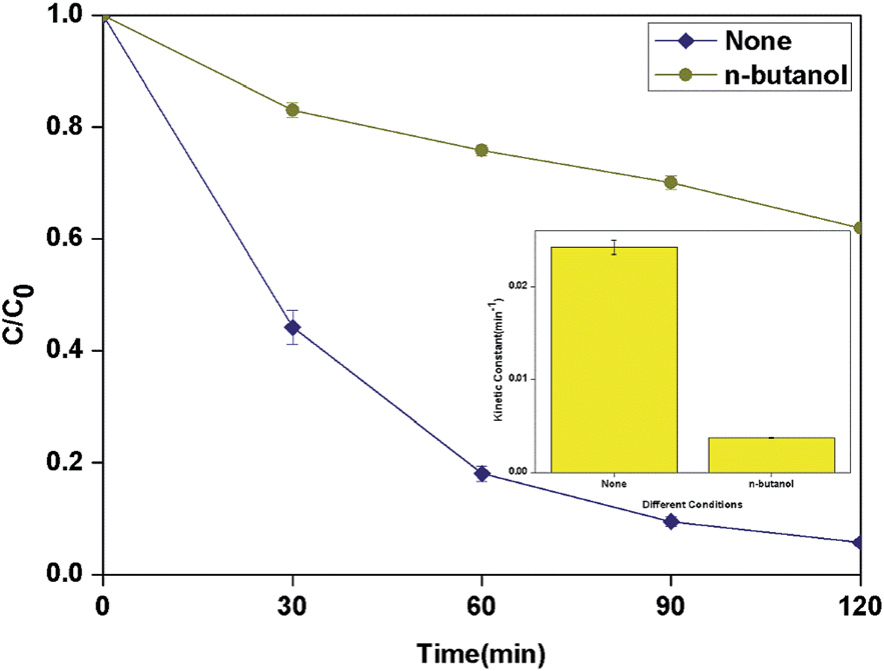
Effect of different radical scavengers in photocatalytic photo-Fenton degradation of BPA at fixed [PLYS-Fe_3_O_4_@TiO_2_] = 1.5 g L^−1^, [H_2_O_2_] = 18.9 mM, [BPA] = 0.088 mM, pH = 7, light intensity = 800 μW cm^−2^, *T* = 25 °C.

According to the results of other researchers, *n*-butanol can react with HO˙ which generated from both surface and bulk solution.^35,36^ The actual reactive species in the process was discriminated by determining the influence of *n*-butanol as radical scavenger on the degradation of BPA. As shown in Fig. 9, 1 M *n*-butanol in solution can scavenge most of the HO˙ produced in the system and the kinetic constant decreased to 3.7 × 10^−3^ min^−1^.

**Fig. 10:**
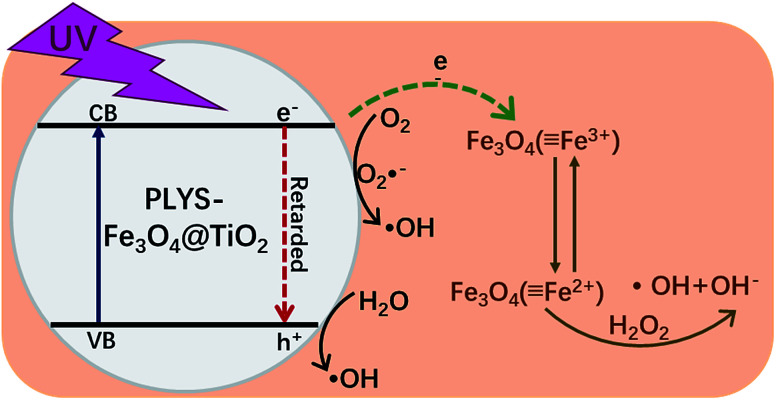
Schematic illustration of the possible mechanism proposed for BPA degradation by PLYS-Fe_3_O_4_@TiO_2_.

Based on all the information obtained above and previous studies by other researchers,^13,37–39^ an assumed mechanism of BPA degradation by PLYS-Fe_3_O_4_@TiO_2_/H_2_O_2_ system is illustrated in Fig. 10. The BPA is mainly removed by HO˙ which was generated from photo-Fenton and photocatalysis process as shown in eqn (3), (14),^40,41^(15)^42,43^ and (16).^13^ Benefiting from the yolk–shell and nanosheet structure, BPA molecules can easily permeate into the surface of Fe_3_O_4_ and degrade by hydroxyl radicals. Most importantly, the photo-induced electrons which generated from TiO_2_ can not only promote the recovery of Fe^2+^ from Fe^3+^, but also inhibit the recombination of electrons and holes.

13 Fe(OH)^2+^ + *hν* → HO˙ + Fe^2+^

14 H_2_O_2_ + *hν* → 2HO˙

15 TiO_2_ + *hν* → e_CB_^−^ + h_VB_^+^

16 H_2_O + h_VB_^+^ → HO˙ + H^+^

### 3.4 Catalytic stability test

**Fig. 11:**
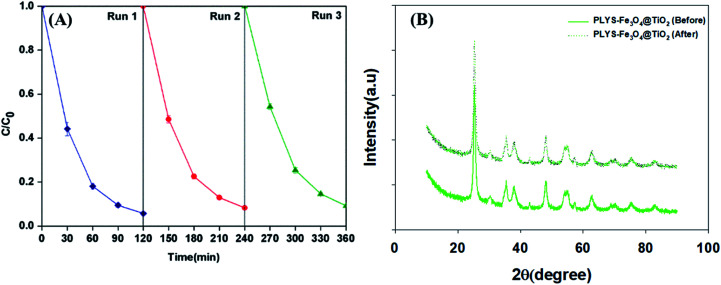
(A) shows the curve of response time in photocatalytic photo-Fenton decomposition, (B) shows the XRD results of the catalyst before and after the reaction. Conditions: [PLYS-Fe_3_O_4_@TiO_2_] = 1.5 g L^−1^, [H_2_O_2_] = 18.9 mM, [BPA] = 0.088 mM, light intensity = 800 μW cm^−2^, *T* = 25 °C, pH = 7.

Recyclability is a crucial factor which can affect the catalyst application in economic perspective.^43–45^ In order to observe the stability, the catalyst was collected by magnetic separation after treatment, washed by deionized water and ethanol respectively, dried at 353 K and was evaluated by BPA degradation under the standard reaction conditions. As shown in Fig. 11(A), the degradation kinetic constant of reused catalyst is 24.2 × 10^−3^, 21.1 × 10^−3^ and 20.3 × 10^−3^ min^−1^ for the first, second and third run, respectively. In addition, the Fe leaching (Fig. S9†) was less than 0.21 mg L^−1^ in the whole process. In addition, XRD analysis before and after the reaction of PLYS-Fe_3_O_4_@TiO_2_ catalyst in Fig. 11(B) showed that the characteristics of the catalyst did not change. The results demonstrated that PLYS-Fe_3_O_4_@TiO_2_ may be used as a promising catalyst for BPA degradation because of good recyclability and stability.

## 4. Conclusion

In summary, pea-like yolk–shell structured PLYS-Fe_3_O_4_@TiO_2_ has been successfully synthesized *via* a combination of kinetics-controlled mechanical force-driven growth and hydrothermal etching assisted crystallization method. And the catalyst was first tested as a heterogeneous photocatalytic photo-Fenton catalyst for BPA degradation, catalyst and H_2_O_2_ dose, initial pH and concentration of BPA are important variables on the degradation process.

The rate constant of BPA degradation in PLYS-Fe_3_O_4_@TiO_2_/H_2_O_2_/UV system was 24.2 × 10^−3^ min^−1^ (pH = 7). The BPA decomposition rate constant decreased with increasing pH. As a result of studying the rate of decomposition reaction according to the initial concentration of BPA, we found that there is an optimal BPA decomposition rate constant value at a certain concentration. Through the XRD analysis after the cycling experiment and before and after the reaction of the catalyst, the activity of the catalyst was still very stable, indicating that the catalyst had excellent stability and reusability. This study may provide useful information to further develop some effective heterogeneous photocatalytic photo-Fenton catalysts for degradation of organic pollutants.

## Conflicts of interest

There are no conflicts to declare.

## Supplementary Material

RA-009-C9RA04084F-s001
