# Supplementary material for: Application of pea-like yolk–shell structured Fe3O4@TiO2 nanosheets for photocatalytic and photo-Fenton oxidation of bisphenol-A
Source: RSC Adv. 2019 Jul 17;9(38):22153–60. doi: 10.1039/c9ra04084f (PMC9066648; doi:10.1039/c9ra04084f)
Supplement: RA-009-C9RA04084F-s001 [file RA-009-C9RA04084F-s001.pdf]

Electronic Supplementary Material (ESI) for RSC Advances.
This journal is © The Royal Society of Chemistry 2019

Application of pea-like yolk-shell structured Fe_3O_4@TiO_2 nanosheets for photocatalytic and photo-Fenton oxidation of bisphenol-A

Xingxing Li, Mingcan Cui, Yonghyeon Lee, Jongbok Choi, Jeehyeong Khim*

School of Civil, Environmental, and Architectural Engineering, Korea University, 145 Anam-ro, Seongbuk-gu, Seoul 02841, Republic of Korea

E-mail: hyeong@korea.ac.kr

List of supporting information

1. Schematic diagram of experimental set up

2.Wave length of the UV lamp

3. TEM images of Fe_3O_4@SiO_2@TiO_2 with different ammonia content

4.TEM images of pea-like Fe_3O_4@SiO_2@TiO_2 with different diameter of mixing paddle

5.Schematic diagram of formation mechanism of pea-like Fe_3O_4@SiO_2@ TiO_2

6.TEM images of PLYS-Fe_3O_4@TiO_2 spheres with different concentration of NaOH

7.TEM and SEM images of PLYS-Fe_3O_4@TiO_2

8. EDS of PLYS-Fe_3O_4@TiO_2

9. Iron leaching test

10. TOC removal efficiency calculation

11. Comparing reaction rate constants in different systems and conditions

1. Schematic diagram of experimental set

Fig. S1

2.Wave length of the UV lamp

Fig. S2

3. TEM images of Fe_3O_4@SiO_2@TiO_2 with different ammonia content

Fig. S3

4. TEM images of pea-like Fe_3O_4@SiO_2@TiO_2 with different diameter of mixing paddle

Fig.S4

5. Formation mechanism of pea-like Fe_3O_4@SiO_2@ TiO_2

The formation of a polymerized TiO_2 via sol-gel process including hydrolysis and condensation process of TIPO is mainly controlled by the concentration of ammonia while maintaining other parameters [1,2]. As shown is **Fig.S5**, there are four models

observed for nucleation and growth of TiO_2 on the surface of Fe_3O_4@SiO_2 spheres under different ammonia concentration.

In the **Model 1**, due to the concentration of ammonia is very low, the hydrolysis and condensation rate of TIPO is so slow that the heterogeneous nucleation is difficult to proceed. Therefore, there is no TiO_2 nanoparticles on the surface of Fe_3O_4@SiO_2 spheres [3]. Increasing the concentration of ammonia slightly, which can promote the hydrolysis and condensation of TIPO, thus the concentration of titanium oligomers increases. When the concentration of titanium oligomers is more than the critical concentration of heterogeneous nucleation, the heterogeneous nucleation of TiO_2 shell produces on the Fe_3O_4@SiO_2 spheres surface (**Model 2**). With the increase of concentration of titanium oligomers which is higher than the supersaturated concentration, the small TiO_2 nuclei can be formed both on the surface of TiO_2 shell and the solution system (**Model 3**). Furthermore, when the initial contents of ammonia are too high, a continual cascading of TiO_2 nuclei in addition to growth occurs *via* diffusion and polymerization of titanium oligomers to the TiO_2 nuclei. Meanwhile, under the mechanical force-driven [4], the pea-like structured Fe_3O_4@SiO_2@TiO_2 particles are formed (**Model 4**).

Fig.S5

6. TEM images of PLYS-Fe_3O_4@TiO_2 spheres with different concentration of NaOH

Fig.S6

7. TEM and SEM images of PLYS-Fe_3O_4@TiO_2

Fig.S7

8. EDS of PLYS-Fe_3O_4@TiO_2

The integrated energy dispersive X-ray spectroscopy (EDS) shows that the content of Ti, Fe, O is 34.35%, 13.17% and 40.61%, respectively.

Fig. S8

9. Iron leaching test

The iron leaching from the PLYS-Fe_3O_4@TiO_2 into the solution were measured using an inductively coupled plasma atomic emission spectrometer (ICP-AES; Perkin Elmer 5300DV).

Fig.S9

10.TOC removal efficiency calculation

The removal percentage of TOC was calculated by using Eq. S1

$$\text{TOC removal}=(1-\frac{TOCt}{TOC0})\times 100\% \qquad (1)$$

where TOC_0 and TOC_t are the TOC values at initial and time of the photocatalytic photo-Fenton process, respectively. As shown in **Fig.S10**, 65.2 % TOC was removed at pH 7 in 2h.

Fig.S10

Table S1

Reference

[1] Livage, J, Henry, M,Sanchez, C. Prog, Solid State Chem 18 (1988) 259-341.

[2] Barringer. E, Bowen, H, Langmuir 1 (1985) 414-420.

[3] W. Li, J. Yang, Z. Wu, J. Wang, B. Li, S. Feng, Y. Deng, F. Zhang, D. Zhao, A versatile kinetics-controlled coating method to construct uniform porous TiO_2 shells for multifunctional core-shell structures, J Am Chem Soc, 134 (2012) 11864-11867.

[4] Y. Tang, Y. Zhang, J. Deng, J. Wei, H. Le Tam, B.K. Chandran, Z. Dong, Z. Chen, X. Chen, Mechanical force-driven growth of elongated bending TiO_2 -based nanotubular materials for ultrafast rechargeable lithium ion batteries, Adv Mater, 26 (2014) 6111-6118.

[5] S. Ahmad Mokhtari, M. Farzadkia, A. Esrafili, R.R. Kalantari, A.J. Jafari, M. Krmani, M. Gholami, Bisphenol A removal from aqueous solutions using novel UV/Persulfate/H_2O_2/Cu system: optimization and modelling with central composite design and response surface methodology, J. Env. Heal. Sci. Eng. (2016) 14-19

[6] M. Saquib, M. Abu Tariq, M.M. Haque, M. Muneer, Photocatalytic degradation of disperse blue 1 using UV/TiO_2/H_2O_2 process, J. Env. Mana. (2008) 88, 300-306

[7] S. Kang, J.Y. Do, S.W. Jo, K.M. Kim, K.M. Jeong, S.M. Park, M. Kang, Efficient removal of bisphenol A by an advanced photocatalytic oxidation-type UV/H_2O_2/Fe-loaded TiO_2 system. Bulletin of the Korean Chemical Society, (2006) 36, 2006-2014

Figure List of supporting information

Fig.S1 Schematic diagram of experimental set up

Fig.S2 Wave length of the UV lamp

Fig.S3 TEM images of Fe_3O_4@SiO_2@TiO_2 with different ammonia content

Fig.S4 TEM images of pea-like Fe_3O_4@SiO_2@TiO_2 with different diameter of mixing paddle

Fig.S5 Schematic diagram of formation mechanism of pea-like Fe_3O_4@SiO_2@TiO_2

Fig.S6 TEM images of Fe_3O_4@tatiate spheres with different concentration of NaOH

Fig.S7 TEM and SEM images of PLYS- Fe_3O_4@TiO_2

Fig.S8 EDS of PLYS- Fe_3O_4@TiO_2

Fig.S9 Iron leaching test

Fig.S10 TOC removal efficiency calculation

Table List of supporting information

Table S1 Comparing reaction rate constants in different systems and conditions

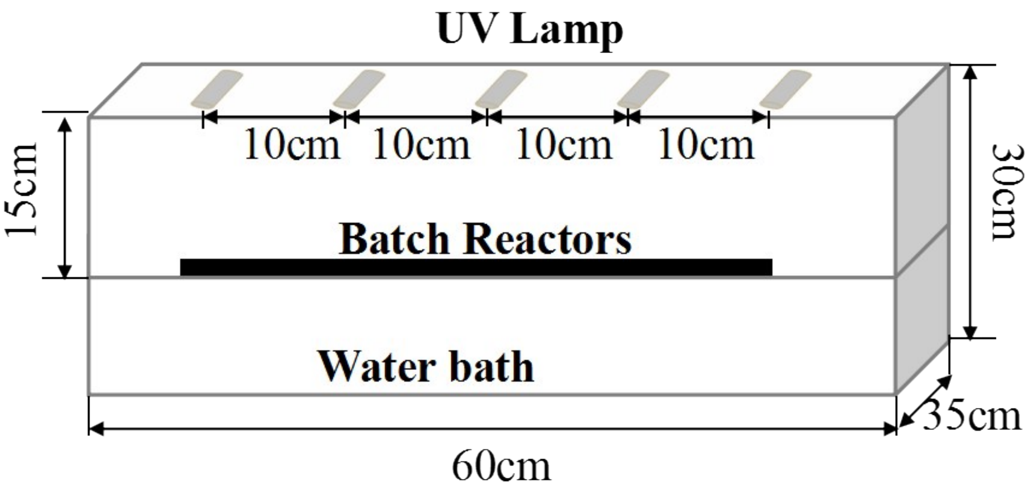

Fig.S1 Schematic diagram of experimental set up

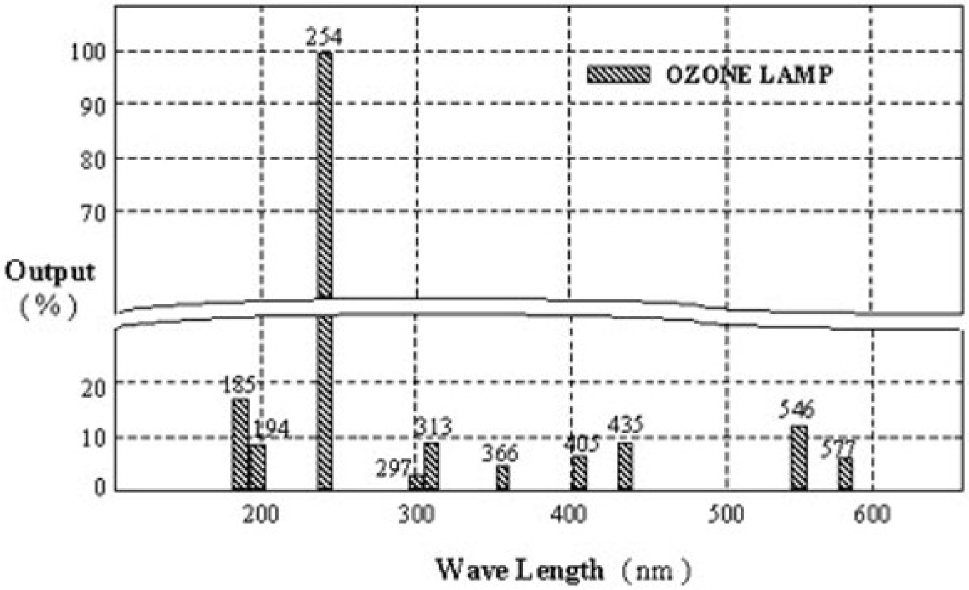

Fig.S2 Wave length of the UV lamp

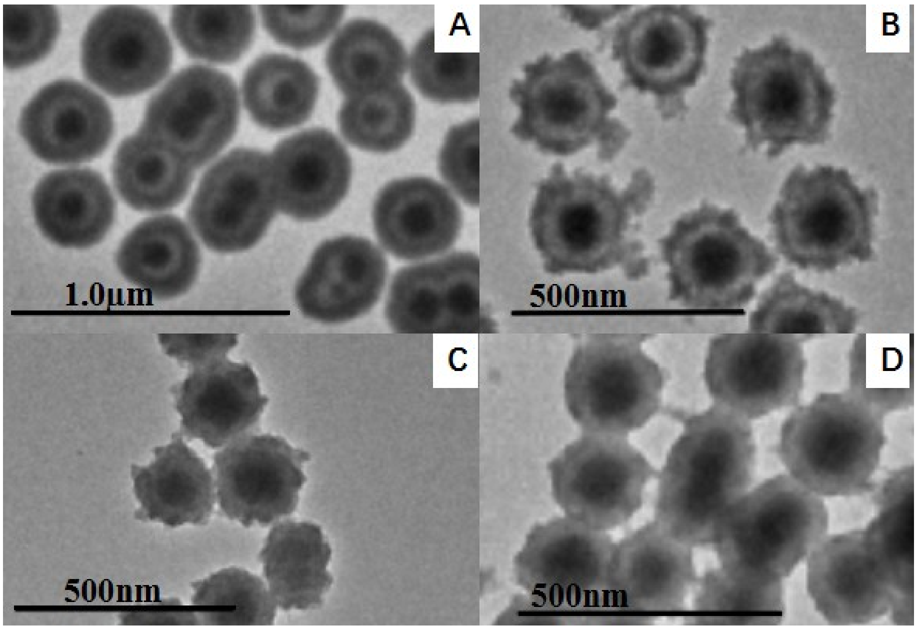

Fig.S3 TEM images of Fe_3O_4@SiO_2@TiO_2 with different ammonia content

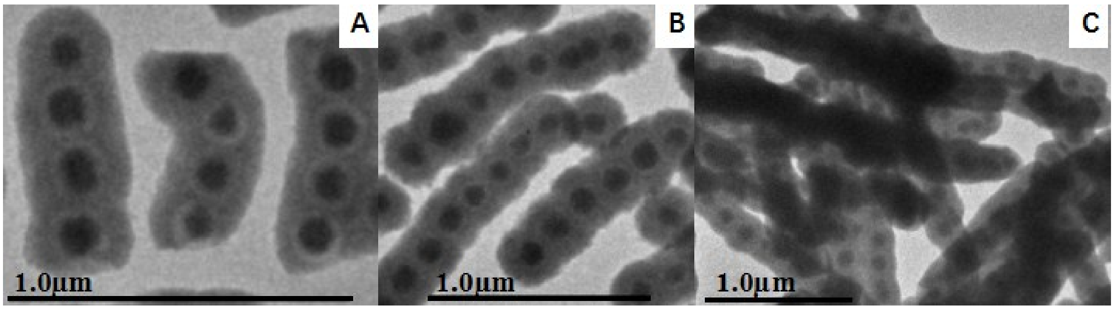

Fig.S4 TEM images of pea-like Fe_3O_4@SiO_2@TiO_2 with different diameter of mixing paddle

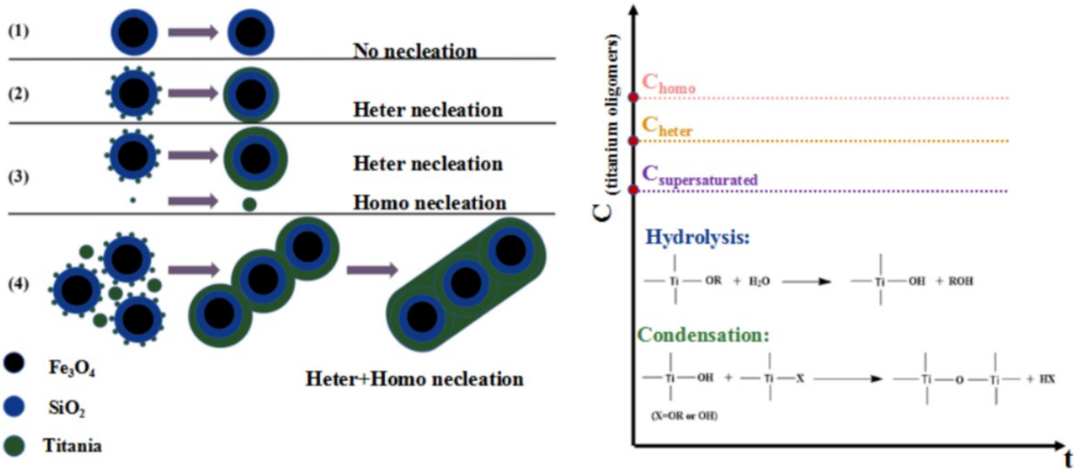

Fig.S5 Schematic diagram of formation mechanism of pea-like Fe_3O_4@SiO_2@TiO_2

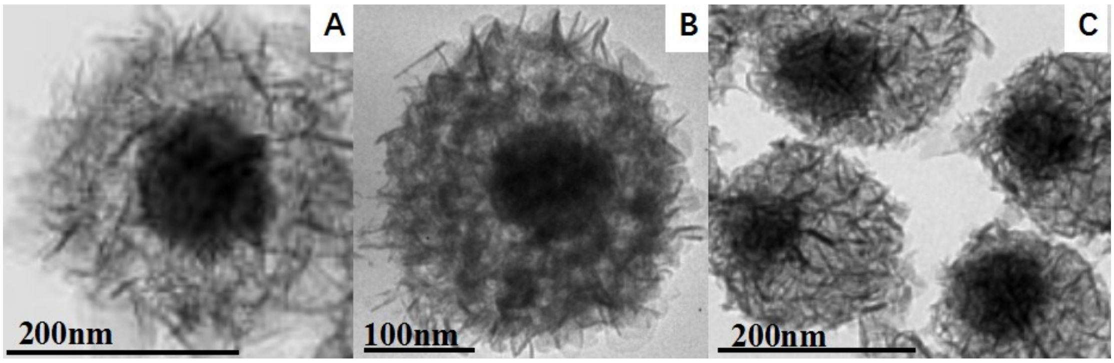

Fig.S6 TEM images of PLYS-Fe_3O_4@TiO_2 spheres with different concentration of NaOH

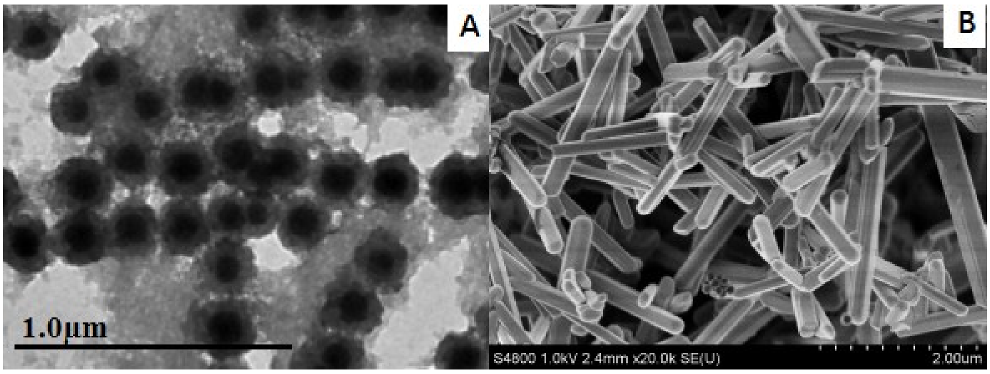

Fig.S7 TEM and SEM images of PLYS-Fe_3O_4@TiO_2

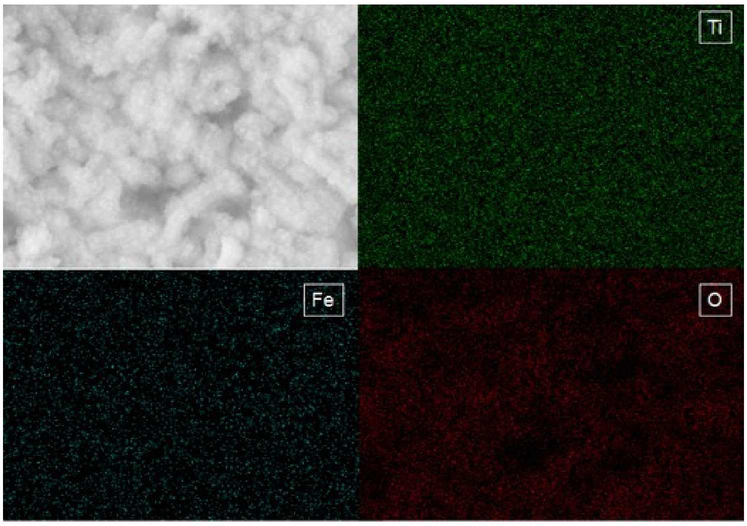

Fig.S8 EDS of PLYS-Fe_3O_4@TiO_2

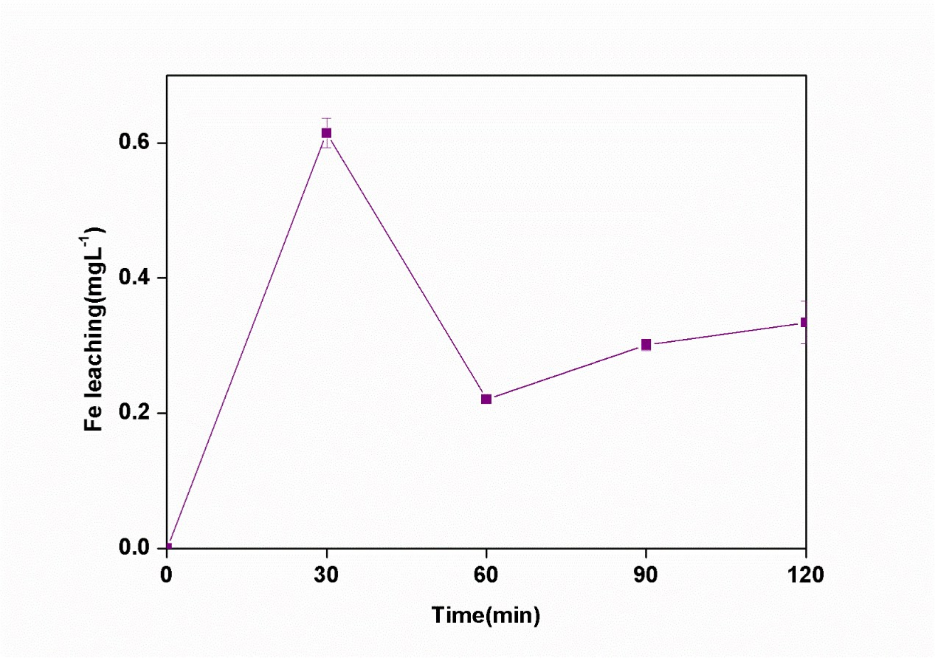

Fig.S9 Iron leaching test

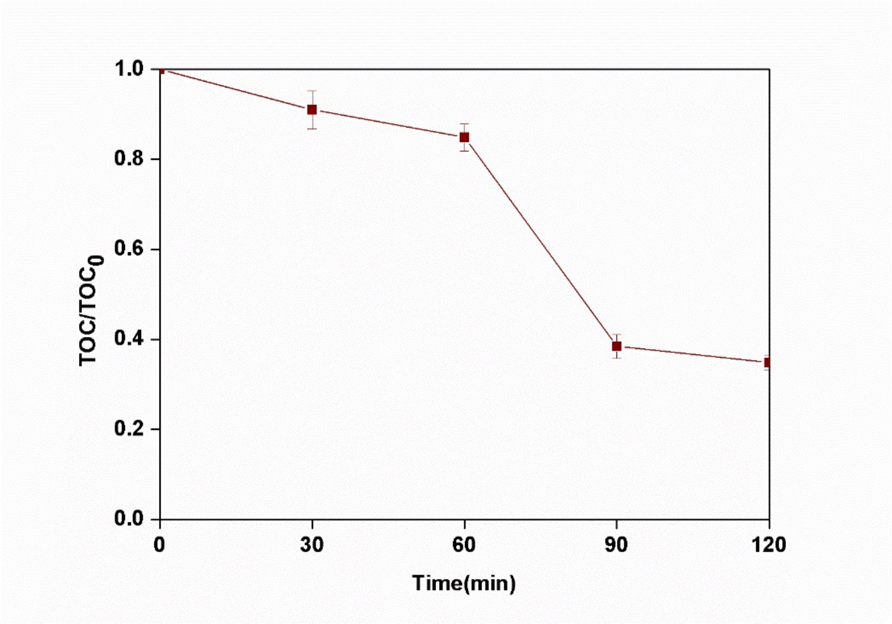

Fig.S10 TOC removal efficiency calculation

Table S1 Comparing reaction rate constants in different systems and conditions

System	Condition and	kinetic constant (min^{-1})	Ref.
Only UV	Intensity=800 μWcm^{-2}, Temp.=25℃, BPA=0.088 mM	ND	
Only PLYS-Fe_3O_4@TiO_2	PLYS-Fe_3O_4@TiO_2=1.5 g L^{-1}, BPA=0.088 mM, Temp.=25℃,	ND	
Only H_2O_2	H_2O_2=18.9 mM, BPA=0.088 mM, Temp.=25℃	1.5×10^{-3}	
UV/H_2O_2	H_2O_2=18.9 mM, BPA=0.088 mM, Temp.=25℃, Intensity=800 μWcm^{-2}	3.0×10^{-3}	
UV/ PLYS-Fe_3O_4@TiO_2	PLYS-Fe_3O_4@TiO_2=1.5 g L^{-1}, BPA=0.088 mM, Temp.=25℃, Intensity=800 μWcm^{-2}	3.4×10^{-3}	
PLYS-Fe_3O_4@TiO_2/H_2O_2	PLYS-Fe_3O_4@TiO_2=1.5 g L^{-1}, BPA=0.088 mM, Temp.=25℃, H_2O_2=18.9 mM	2.7×10^{-3}	This study
UV/TiO_2(P25)/H_2O_2	Intensity=800 μWcm^{-2}, TiO_2(P25)=1.5 g L^{-1}, BPA=0.088 mM, Temp.=25℃, H_2O_2=18.9 mM	11.5×10^{-3}	
UV/Fe_3O_4/H_2O_2	Intensity=800 μWcm^{-2}, Fe_3O_4=1.5 g L^{-1}, BPA=0.088 mM, Temp.=25℃, H_2O_2=18.9 mM	2.9×10^{-3}	
UV/PLYS-Fe_3O_4@TiO_2/H_2O_2	Intensity=800 μWcm^{-2}, PLYS-Fe_3O_4@TiO_2=1.5 g L^{-1}, H_2O_2=18.9 mM, BPA=0.088 mM, Temp.=25℃	24.2×10^{-3}	
UV/$S_2O_8^{2-}$/H_2O_2/Cu<br>UV/H_2O_2	UV=Mercury lamp (diameter 8×length 90 cm); power=12 W;	43×10^{-3}<br>6×10^{-3}	[5]

UV/Cu	BPA=0.099 mM; $S_2O_8^{2-}$=0.339	3.4×10^{-3}	
UV/S_2O_2/H_2O_2	mM; H_2O_2=0.294 mM;	18×10^{-3}	
UV/H_2O_2/Cu	Cu^{+}=0.273 mM	9.6×10^{-3}	
UV/$S_2O_8^{2-}$/Cu		11×10^{-3}	
UV/Fe-TiO_2(3% Fe)/H_2O_2	UV=Mercury lamp(diameter 2.0×length 15 cm); Intensity=18 W cm^{-2}; BPA=0.044 mM; H_2O_2=1 mL(2L volume); catalytic loading=1 g L^{-1}	15.4×10^{-3}	[6]
			[7]
UV/TiO_2(P25)/H_2O_2	UV=254 nm(1.25 mW cm^{-2});	5.7×10^{-3}	
UV/TiO_2	dye=0.25 mM; H_2O_2=0.1 mL(250 mL); TiO_2=1g L^{-1}	3.1×10^{-3}	
